# Sensitizing *Clostridium difficile* Spores With Germinants on Skin and Environmental Surfaces Represents a New Strategy for Reducing Spores via Ambient Mechanisms

**DOI:** 10.20411/pai.v2i3.221

**Published:** 2017-10-30

**Authors:** Michelle M. Nerandzic, Curtis J. Donskey

**Affiliations:** 1 Research Service, Veterans Affairs Medical Center, Cleveland, Ohio; 2 Case Western Reserve University School of Medicine, Cleveland, Ohio; 3 Geriatric Research, Education and Clinical Center, Veterans Affairs Medical Center, Cleveland, Ohio

**Keywords:** *Clostridium difficile*, spores, disinfection, hand hygiene, germination

## Abstract

**Background:**

*Clostridium difficile* is a leading cause of healthcare-associated infections worldwide. Prevention of *C. difficile* transmission is challenging because spores are not killed by alcohol-based hand sanitizers or many commonly used disinfectants. One strategy to control spores is to induce germination, thereby rendering the spores more susceptible to benign disinfection measures and ambient stressors.

**Methods/Results:**

*C. difficile* spores germinated on skin after a single application of cholic acid-class bile salts and co-germinants; for 4 *C. difficile* strains, recovery of viable spores from skin was reduced by ~0.3 log_10_CFU to 2 log_10_CFU after 2 hours and ~1 log_10_CFU to > 2.5 log_10_CFU after 24 hours. The addition of taurocholic acid to 70% and 30% ethanol significantly enhanced reduction of viable spores on skin and on surfaces. Desiccation, and to a lesser extent the presence of oxygen, were identified as the stressors responsible for reductions of germinated spores on skin and surfaces. Additionally, germinated spores became susceptible to killing by pH 1.5 hydrochloric acid, suggesting that germinated spores that remain viable on skin and surfaces might be killed by gastric acid after ingestion. Antibiotic-treated mice did not become colonized after exposure to germinated spores, whereas 100% of mice became colonized after exposure to the same quantity of dormant spores.

**Conclusions:**

Germination could provide a new approach to reduce *C. difficile* spores on skin and in the environment and to render surviving spores less capable of causing infection. Our findings suggest that it may be feasible to develop alcohol-based hand sanitizers containing germinants that reduce spores on hands.

**STANDFIRST**

Spore germination could provide a new approach to reduce the burden of *C. difficile* spores on skin and in the environment, and decrease the likelihood that ingested spores will cause disease.

## INTRODUCTION

*Clostridium difficile* is a spore-forming anaerobic bacterium that is the most common cause of healthcare-associated diarrhea in developed countries [1, 2]. In the United States, *C. difficile* causes an estimated 453,000 infections each year and is associated with approximately 29,000 deaths [3]. Consequently, *C. difficile* has been classified as an urgent threat level pathogen by the Centers for Disease Control and Prevention [4].

Prevention of *C. difficile* transmission is challenging, in part because the spores are not killed by alcohol-based hand sanitizers, antimicrobial soaps, and many commonly used surface disinfectants [5–7]. Spores on the skin of patients and healthcare workers present a particular challenge because sporicidal disinfectants are not safe for use on skin, and soap and water hand washing is only modestly effective in reducing spore contamination [5–7]. The lack of effective strategies to remove spores from skin is of critical importance because healthcare workers frequently contaminate their hands with spores when caring for patients with *C. difficile* infection (CDI) or asymptomatic carriage of toxigenic *C. difficile* [8, 9]. Recent evidence that asymptomatic carriers contribute to transmission is concerning because carriers outnumber CDI cases, but they remain unrecognized. Thus, there is an urgent need for development of safe and effective strategies to reduce spore contamination on skin and surfaces.

Sporicidal strategies are impeded by the highly-resistant dormant spore structure consisting of multiple proteinaceous layers surrounding a dehydrated core [10, 11]. Spores remain dormant until they are exposed to agents that trigger them to germinate [12, 13]. The germination mechanism of *C. difficile* is initiated by the presence of cholic acid-class bile salts and a nutrient co-germinant, leading to a cascade of events that causes the spore's cortex layer to deteriorate and eventually the spore core to re-hydrate [12, 13]. Because germinated spores have increased susceptibility to stressors such as radiation and heat, induction of germination is a potential strategy to control spore-forming organisms [14, 15]. Wheeldon *et al* reported that exposure of *C. difficile* spores to the bile salt taurocholate under aerobic conditions enhanced killing on copper surfaces [16]. We recently reported that quaternary ammonium solutions containing germinants reduced environmental contamination by sensitizing *C. difficile* spores, leaving them susceptible to ambient room conditions and enhancing killing by technologies such as blue light and UV-C radiation [17].

There are no published reports in which triggering of germination has been studied as a strategy for reducing spores on skin. Here, we used a porcine skin model to test the hypothesis that application of germinants alone or in an ethanol matrix would sensitize *C. difficile* spores to ambient stressors. We examined the role of desiccation and ambient oxygen in the killing of sensitized spores. Finally, we examined susceptibility of germinated spores to killing by acid and tested whether germination results in the reduced ability of spores to establish colonization in antibiotic-treated mice. Our findings suggest that germination could provide a new approach to reduce the burden of *C. difficile* spores on skin and in the environment and to decrease the likelihood that ingested spores will cause disease.

## METHODS

### Spore strains and growth conditions.

R20291 and VA 17 are hypervirulent BI/NAP1/027 epidemic (cdtB+) strains and VA 11 is a non-epidemic (cdtB-) REA J strain. ATCC 43598 is a toxigenic (*tcdA*+, *tcdB*+) strain classified toxinotype VIII/ribotype 017 from serogroup F. ATCC 43593 is a non-toxigenic (tcdA-, tcdB-) strain classified ribotype 060 from serogroup B. Cultures of *C. difficile* were incubated at 37°C for 48 hours in a Whitley MG1000 anaerobic workstation (Microbiology International, Frederick, MD) on pre-reduced cycloserine cefoxitin-brucella agar containing 0.1% taurocholic acid and lysozyme 5 mg/L (CDBA) [18].

### Preparation of spores.

*C. difficile* spores were prepared as previously described by Perez *et al* [19] with the following modifications. Spores were harvested from the Clospore media after 2 weeks of incubation in a Whitley MG1000 anaerobic workstation (Microbiology International, Frederick, MD). Vegetative material was removed by density gradient centrifugation in Histodenz (Sigma Aldrich, St. Louis, MO). Prior to testing, spore preparations were confirmed by phase contrast microscopy and malachite green staining to be > 99% dormant, bright-phase spores.

### Recovery of Clostridium difficile spores from an ex vivo porcine skin model after treatment with germinants.

The *ex vivo* porcine skin model is a widely adopted analogue for human skin in dermatological studies [20]. It is not known whether spores can germinate in the complex environment of the skin or if germination will result in reduced spore survival on skin. Here we determined whether topical application of cholic acid-class bile salts with or without a mineral or amino acid co-germinant would be effective for stimulating germination of *C. difficile* spores on skin and reducing survival. Glycine has been well-defined as a co-germinant with bile salts, but more recently it has been suggested that exogenous calcium chloride is also an effective co-germinant [21].

Sections of porcine skin (1cm^2^) were inoculated with 7 log_10_ colony forming units (CFU) of R20291, VA17, ATCC 43598, and ATCC 43593 spores. Fifty microliters of either water, 70% ethanol, 10mM cholic acid-class bile salt (cholic acid, glycocholic acid, and taurocholic acid), 100mM co-germinant (glycine or calcium chloride), or 10mM bile salt with 100mM co-germinant was applied once and rubbed into each skin section until absorbed. After 2 or 24 hours of incubation at room temperature, the spores were recovered from the skin sections with Dey Engley neutralizer (Becton, Dickinson and Company, Franklin Lakes, NJ) as described by the American Society for Testing and Materials (ASTM) in “Standard Quantitative Carrier Test Method to Evaluate the Bactericidal, Fungicidal, Mycobactericidal, and Sporicidal Potencies of Liquid Chemicals” [22]. The log_10_CFU of viable spores was quantified by the serial drop-plate method on CDBA recovery medium. Germinated spores were identified by heat shocking the recovered spores at 80°C for 5 minutes to determine the heat susceptible fraction. Each experiment was performed 3 times.

### Recovery of Clostridium difficile spores from an ex vivo porcine skin model after treatment with germinants in an ethanol matrix.

Sections of porcine skin (1cm^2^) were inoculated with 7 log_10_CFU of R20291 spores. Fifty micro-liters of water, 70% ethanol, 70% ethanol containing 10mM chenodeoxycholic acid, germinant solution (10mM cholic acid-class bile salt and 100mM calcium chloride), or germinant solution suspended in 70% ethanol was applied once and rubbed into each skin section until absorbed. The porcine skin sections were processed and quantified as described above in *Recovery of Clostridium difficile spores from an ex vivo porcine skin model after treatment with germinants* 24 hours post-incubation at room temperature. Each experiment was performed 3 times.

### Exposure of C. difficile spores to germinants on environmental surfaces.

Two sets of stainless steel carriers (1cm^2^) were placed on a hospital bedside table and inoculated with 6 log_10_CFU of R20291, VA17, ATCC 43598, and VA11 spores. Both sets of carriers were sprayed until uniformly wet with either water, 30% or 70% ethanol, germinant solution (10mM taurocholic acid/100mM calcium chloride), or germinant solution suspended in 30% or 70% ethanol. The surfaces dried approximately 10 minutes after spraying. One set of carriers was processed and quantified as described above 24 hours post-incubation at room temperature. After 24 hours, the second set of carriers (VA17 and VA11) was exposed to another uniform application of test solutions and allowed to air dry for an additional 24 hours, then the spores were recovered from the carriers and the log_10_CFU of viable and germinated spores was determined. The 2 experiments were performed in triplicate.

### The impact of environmental stressors on reduction of germinated Clostridium difficile spores.

Eight log_10_ CFU of VA17 spores were exposed to 1 mL of germinants (10mM taurocholic acid and 100mM calcium chloride) or water (baseline) in a test tube for 30 minutes under ambient conditions (22°C, aerobic conditions). Spore suspensions were washed 3 times and then 10 μL (6 log_10_CFU) was inoculated into the empty well of a glass slide (dry conditions) or a well containing nutrient free agar (moist conditions). The glass slides were placed on the bench top (aerobic conditions) or in an anaerobe containment system (anaerobic conditions) for 24 hours. The temperature was maintained at 22°C for the duration of the experiment. The spores were recovered from the slides by applying 100 μL of Dey Engley neutralizer to each well and transferring contents to a microcentrifuge tube. For wells containing agar, mild heat (50°C for 5 minutes) was applied before performing quantifying assays to de-solidify the agar and assure spores were in suspension. Recovery of viable spores was quantified by the serial drop-plate method on CDBA recovery medium. Mean log_10_CFU reduction was determined by comparing the recovery of water-treated spores with the recovery of germinant-treated spores. Each experiment was performed 3 times.

### Susceptibility of germinated spores to pH 1.5 HCl.

One thousand colony forming units of VA17, VA11, and ATCC 43598 spores were exposed to 1 mL of germinants (10mM taurocholic acid and 100mM calcium chloride) in a test tube for 1, 3, 5, 10, or 30 minutes under ambient conditions (22°C, aerobic conditions). At the appropriate time point, spores were washed 3 times with water to remove germinants and then resuspended in 1 mL of pH 1.5 hydrochloric acid for 10 minutes. Spore suspensions were neutralized with phosphate buffered saline (PBS) pH 7.5 and washed 3 times to remove hydrochloric acid. After the final wash, the spore pellet was suspended in 100 μL of PBS and the contents were spread on a plate of CDBA recovery medium to enumerate recovery of viable spores. Each experiment was performed 3 times.

### Exposure of antibiotic-treated mice to germinated and dormant *C. difficile* spores.

The Animal Care Committee of the Cleveland Veterans Affairs Medical Center approved the following experimental protocol. Forty female CF-1 mice weighing about 30 g each (Harlan Sprague-Dawley, Indianapolis, Indiana) were treated with sub-cutaneous injections (0.2 ml total volume) of clindamycin (1.4 mg). Baseline stool samples were collected from each mouse to determine if they were free of *C. difficil*e prior to exposure to spores. Twenty-four hours after antibiotic treatment, the mice were fasted for 1.5 hours to empty their stomachs and stimulate acid production. The mice were separated into 4 empty cages inoculated with either 10 CFU/cm^2^ germinated spores, 10 CFU/cm^2^ dormant spores, 1 CFU/cm^2^ dormant spores, or 0.1 CFU/cm^2^ dormant spores. After 2 hours of exposure, the mice were labeled and placed into individual cages. Stool samples were collected 48 and 72 hours after exposure to spores. *C. difficile* colonization was determined by plating stool samples on CDBA selective agar and the percentage of positive spores was reported (limit of detection was ≥2 log_10_CFU/gram stool).

Germinated and dormant spores were prepared by exposing 9 log_10_CFU of spores to 10mM taurocholic acid and 100mM glycine for 30 minutes at room temperature. Spores were rinsed 3x with cold phosphate buffered saline (PBS) to remove germinants and then suspended in 2 mL of 20% histodenz and layered onto 20 mL of 50% histodenz in a centrifuge tube. The gradient was centrifuged at 15,000*g* for 15 minutes. The top layer (germinated spores) and pellet (dormant spores) were carefully collected and rinsed 5 times with cold PBS. The top layer of spores collected were confirmed by phase contrast microscopy and heat shock at 80°C for 5 minutes to have > 99.99% germination. Similarly, the spore pellet was confirmed to be > 99.9% dormant.

To determine recovery of spores from inoculated cages, 16 cages were prepared with each of the 4 concentrations of inoculum and swabbed at 0, 30, 60, and 120 minutes post inoculation. Swab samples were transferred to pre-reduced CDBA plates to quantify viable colony forming units.

## RESULTS

**Figure 1.**
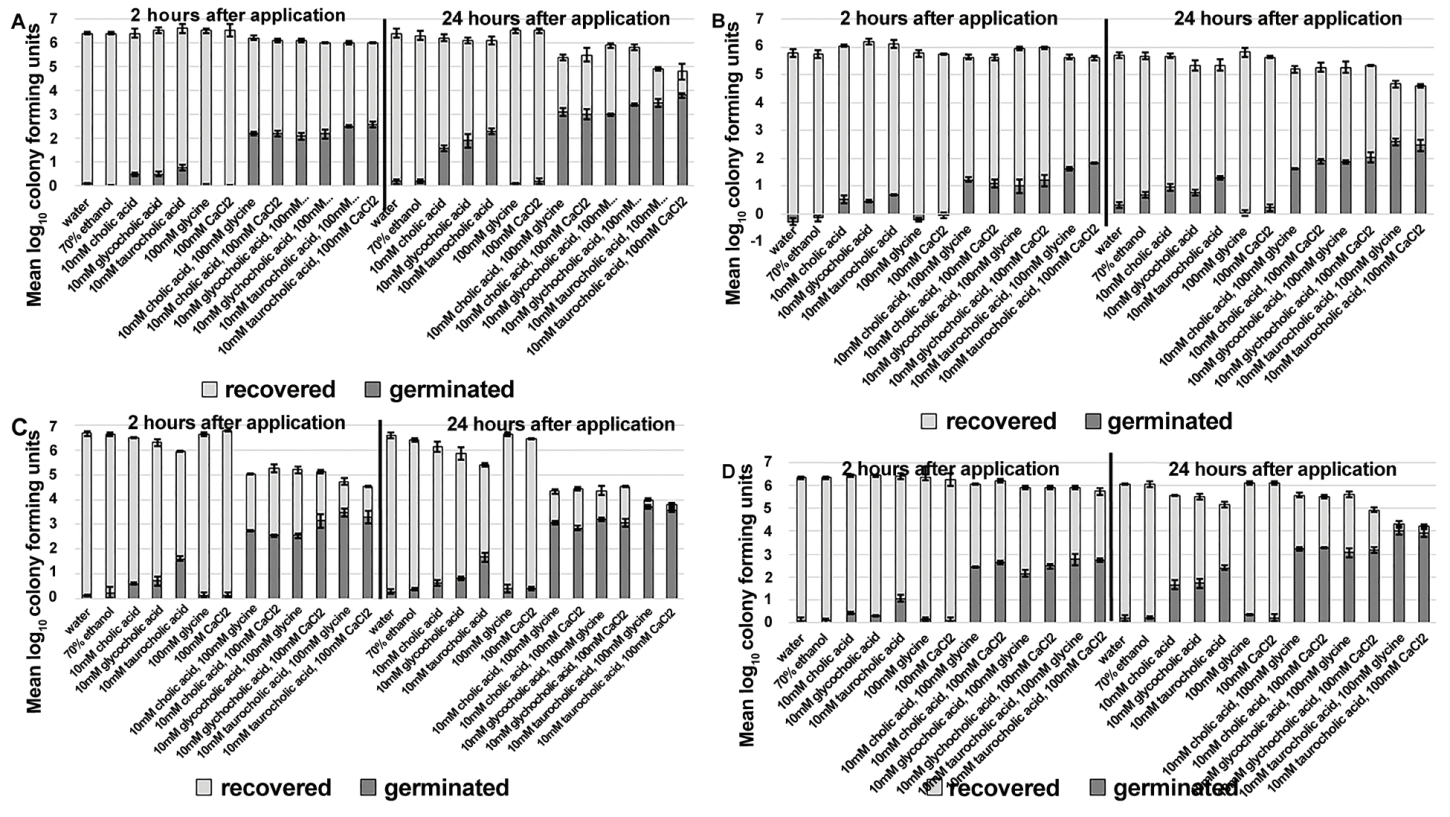
**Recovery of *Clostridium difficile* spores from an *ex vivo* porcine skin model is reduced after sensitizing spores with a single application of germinants.** Sections of porcine skin (1 cm^2^) were inoculated with 7 log_10_ colony forming units (CFU) of *C. difficile* R20291 (A), VA17 (B), ATCC 43598 (C), and ATCC 43593 (D). Then 50 μL of test solution was applied once and rubbed into each skin section until absorbed. After 2 or 24 hours of incubation at room temperature, the spores were recovered from skin sections and the log_10_CFU of viable and germinated spores was determined. The means of data from triplicate experiments are presented. Error bars indicate standard error.

Recovery of *Clostridium difficile* spores from an *ex vivo* porcine skin model was reduced after sensitizing spores with a single application of germinants. The application of cholic acid-class bile salts alone was effective for stimulating some spore germination on skin; however, the addition of a co-germinant significantly increased germination (Figure 1A-D, *P* < 0.01 for each comparison). There was strain-specific variability in the extent of germination, but for each strain tested an increase in the fraction of germinated spores correlated with a decrease in spore survival on skin. Two hours after a single application of bile salts and co-germinants, ~1 log_10_CFU (VA17) to 3 log_10_CFU (ATCC 43598) of spores germinated on the skin and recovery of viable spores from skin was reduced by ~0.3 log_10_CFU to 2 log_10_CFU, respectively. Twenty-four hours after germinant application, ~2–4 log_10_CFU of spores had germinated and recovery of viable spores was reduced by ~1 log_10_CFU to > 2.5 log_10_CFU. Taurocholic acid was superior to cholic or glycocholic acid for inducing germination of spores on skin, however, there was no significant difference whether the co-germinant was glycine or calcium chloride.

Reduction of *Clostridium difficile* spores is enhanced in an *ex vivo* porcine skin model by applying germinants in an ethanol matrix. Due to their efficacy and convenience, alcohol-based hand sanitizers (ie, typically 60%–80% ethanol or isopropanol) have become the primary method of hand hygiene in healthcare settings [23–26]. Unfortunately, alcohols do not have activity against bacterial spores. Therefore, we determined whether topical application of germinants suspended in an ethanol matrix would be effective for triggering spore germination on skin.

**Figure 2.**
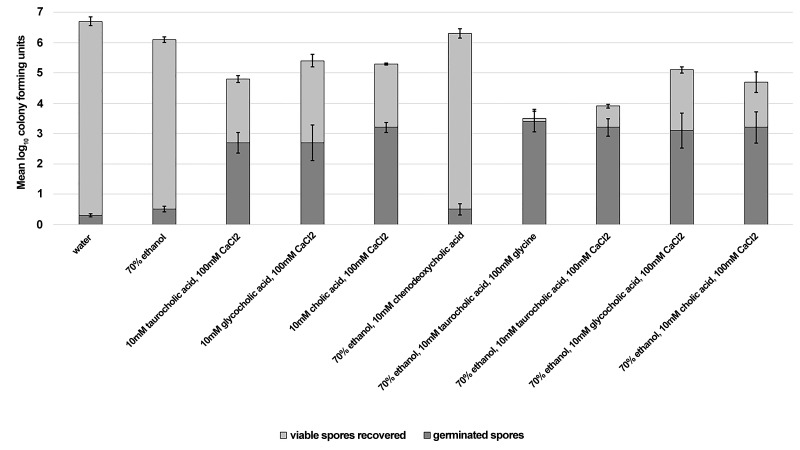
**Reduction of *Clostridium difficile* spores is enhanced in an *ex vivo* porcine skin model by applying germinants in an ethanol matrix.** Sections of porcine skin (1 cm^2^) were inoculated with 7 log_10_ colony forming units (CFU) of *C. difficile* R20291. Then 50 μL of test solution was applied once and rubbed into each skin section until absorbed. After 24 hours of incubation at room temperature, the spores were recovered from skin sections and the log_10_CFU of viable and germinated spores was determined. The means of data from triplicate experiments are presented. Error bars indicate standard error.

Figure 2 shows that *C. difficile* spores germinated on skin when exposed to germinants suspended in a 70% ethanol matrix. Ethanol significantly enhanced reduction of viable spores on skin by 1 log_10_CFU for solutions containing taurocholic acid (*P* < 0.01). Reduction of viable spores on skin was modestly enhanced by the presence of ethanol for glycocholic and cholic acid containing solutions, however, the reductions were not statistically significant. The extent of germination was not enhanced by ethanol-containing solutions, suggesting that ethanol acts as a secondary environmental stressor not a germination enhancer.

### Exposure to germinants reduces recovery of viable Clostridium difficile spores on environmental surfaces.

Non-sporicidal disinfectants such as quaternary ammonium compounds are commonly used in hospitals because they are inexpensive, well-tolerated by staff, and they have good surface compatibility. In a recent study, we found that quaternary ammonium solutions containing germinants resulted in germination in air at room temperature and enhanced killing of *C. difficile* spores by adjunctive disinfection measures [17]. Here, we determined whether germinants delivered in high (70% v/v) and low (30%v/v) concentrations of ethanol solutions would similarly trigger germination of spores on environmental surfaces.

**Figure 3.**
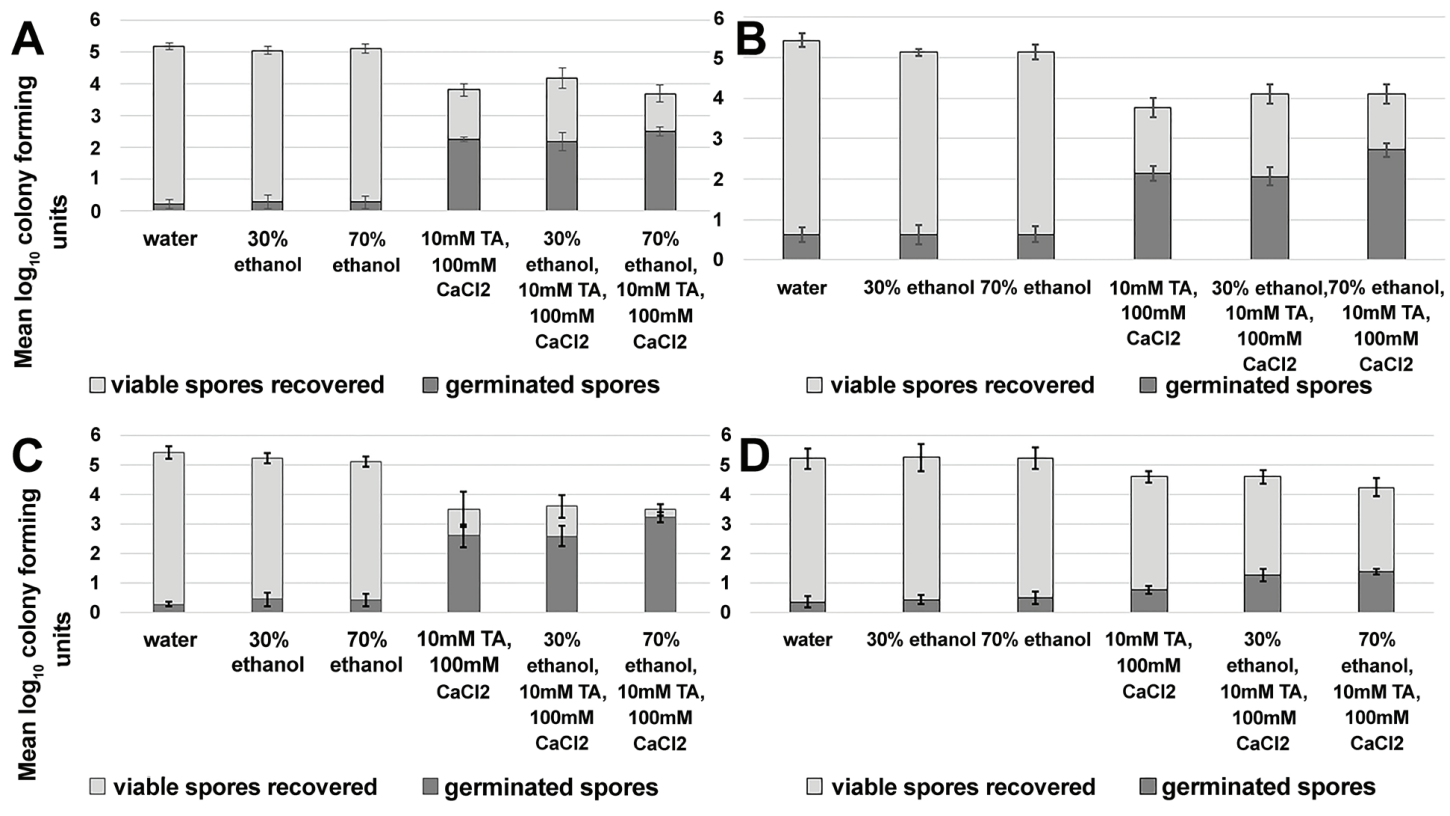
**Exposure to germinants reduces recovery of viable *Clostridium difficile* spores on environmental surfaces.** Stainless steel carriers (1 cm^2^) were placed on a hospital bedside table and inoculated with 6 log_10_ colony forming units (CFU) of *C. difficile* R20291 (A), VA17 (B), ATCC 43598 (C), and VA11 (D). Carriers were sprayed until uniformly wet with test solutions and allowed to air dry. After 24 hours of incubation at room temperature, the spores were recovered from carriers and the log_10_CFU of viable and germinated spores was determined. The means of triplicate data from 2 experiments are presented (N = 6). Error bars indicate standard error.

Spores germinated on surfaces sprayed with a single application of germinant-containing solution, with or without the presence of ethanol (Figure 3A-D). As described previously in the text for germination on skin, there was strain-specific variability in the extent of germination, but for each strain tested an increase in the fraction of germinated spores was correlated with decreased spore survival. Twenty-four hours after spraying with germinant-containing solutions, spores were reduced by 0.6 to > 2 log_10_CFU, depending on the strain tested (VA11 < R20291 < VA17 < ATCC 43598).

**Figure 4.**
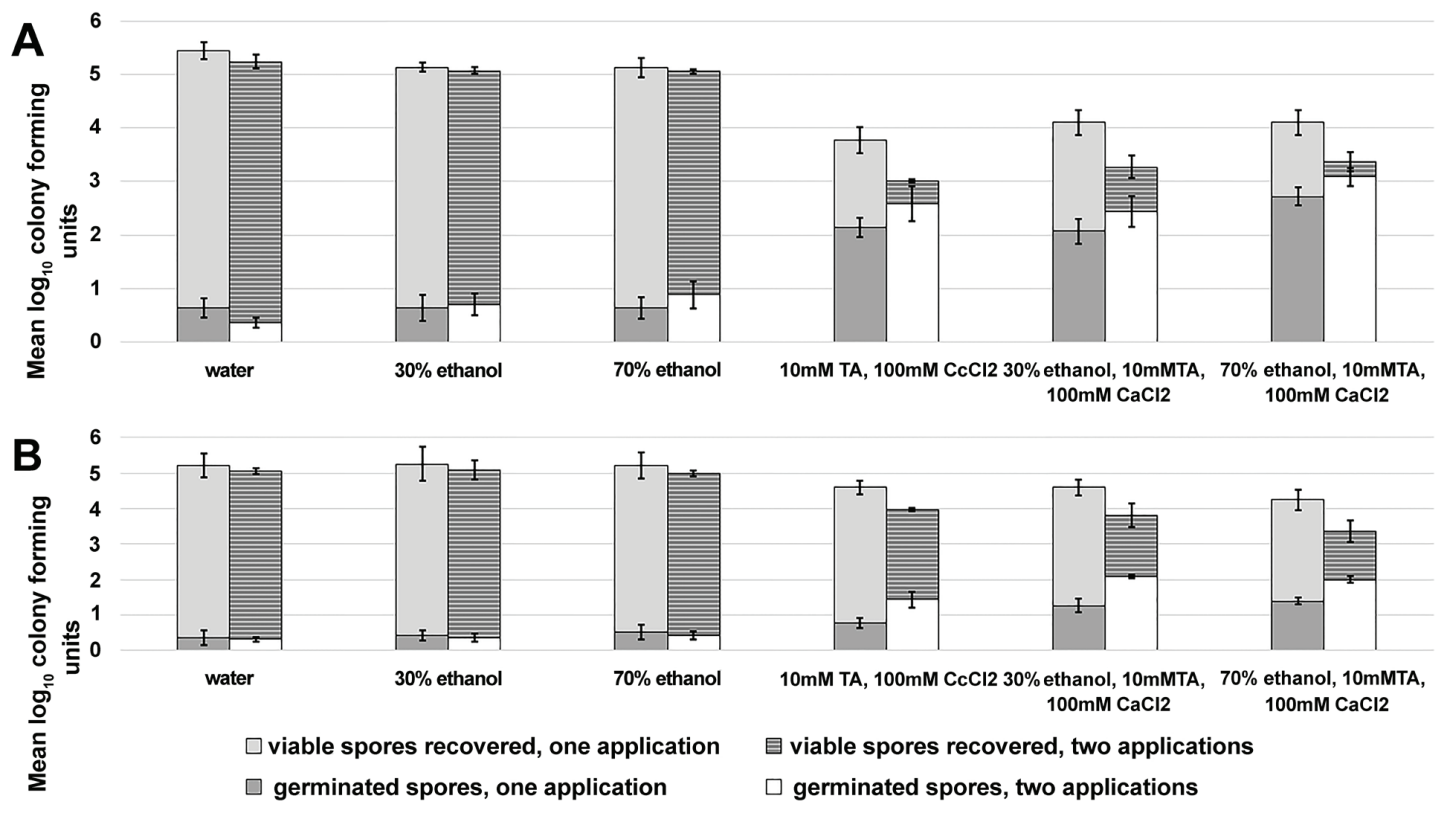
**A second application of germinant solution enhances the reduction of *Clostridium difficile* spores on environmental surfaces.** Two sets of stainless steel carriers (1cm^2^) were placed on a hospital bedside table and inoculated with 6 log_10_ colony forming units (CFU) of *C. difficile* VA17 (A) and VA11 (B). Both sets of carriers were sprayed until uniformly wet with test solutions and allowed to air dry. After 24 hours of incubation at room temperature, 1 set of carriers was collected and the log_10_CFU of viable and germinated spores was determined. The second set of carriers was exposed to another uniform application of test solutions and allowed to air dry, then 24 hours post application the spores were recovered from the carriers and the log_10_CFU of viable and germinated spores was determined. The means of triplicate data from 2 experiments are presented (N = 6). Error bars indicate standard error.

Additionally, Figure 4 shows that a second spray of germinant-containing solution enhances the reduction of VA17 and VA11 spores on surfaces by > 0.5 log_10_CFU for each strain (*P* < 0.01, a single versus 2 applications).

### The impact of environmental stressors on reduction of germinated Clostridium difficile spores.

Germinated spores are more susceptible to stressors than their dormant counterparts [10, 11]. In the previous experiments, spores exposed to germinants were reduced on skin and environmental surfaces. However, the mechanism of spore killing was not known. Here, we provide evidence that desiccation and the presence of oxygen are the stressors responsible for reduction of germinated spores.

**Figure 5.**
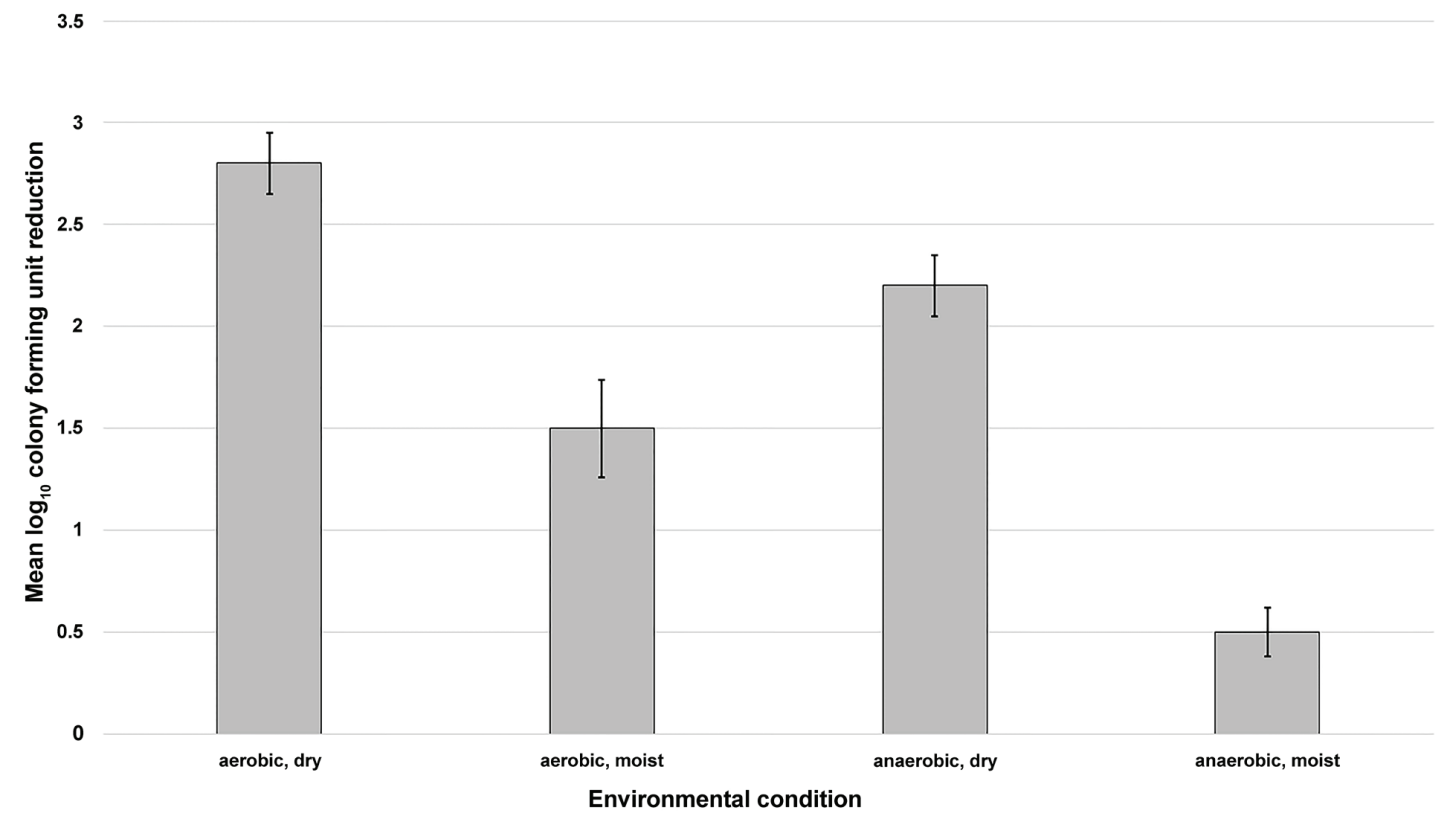
**The impact of environmental stressors on reduction of germinated *Clostridium difficile* spores.** Eight log_10_ colony forming units (CFU) of *C. difficile* VA17 spores were exposed to 1 mL of germinants in a test tube for 30 minutes under ambient conditions (22°C, aerobic). Germinated spores were washed 3 times and then 10 μL (6 log_10_CFU) of the spore suspension was inoculated into the empty well of a glass slide (dry conditions) or a well containing nutrient free agar (moist conditions). The glass slides were placed on the bench top (aerobic conditions) or in an anaerobe containment system (anaerobic conditions) for 24 hours. The temperature was maintained at 22°C for the duration of the experiment. After incubation, the spores were recovered from the slides and the log_10_CFU reduction of spores was determined. The means of data from triplicate experiments are presented. Error bars indicate standard error.

Figure 5 shows that germinated spores are relatively stable after 24 hours if kept moist and free of oxygen. When oxygen was introduced to germinated spores that were kept moist, spore death was increased by 1 log_10_CFU. Germinated spores incubated under oxygen-free conditions but allowed to desiccate were reduced by > 1.5 log_10_CFU, suggesting desiccation plays a greater role in the mode of spore killing. However, both the presence of oxygen and desiccation play a role in spore killing, because germinated spores were reduced by > 2 log_10_CFU under dry, aerobic conditions.

**Germinated spores are susceptible to killing by pH 1.5 hydrochloric acid.** Fractions of germinated spores can remain viable on skin and surfaces for greater than 24 hours. In this study we show that germinated spores are susceptible to pH 1.5 hydrochloric acid (normal gastric pH is 1.5–3.5), suggesting that viable germinated spores remaining on skin and surfaces may be killed by stomach acid if they are ingested.

**Figure 6.**
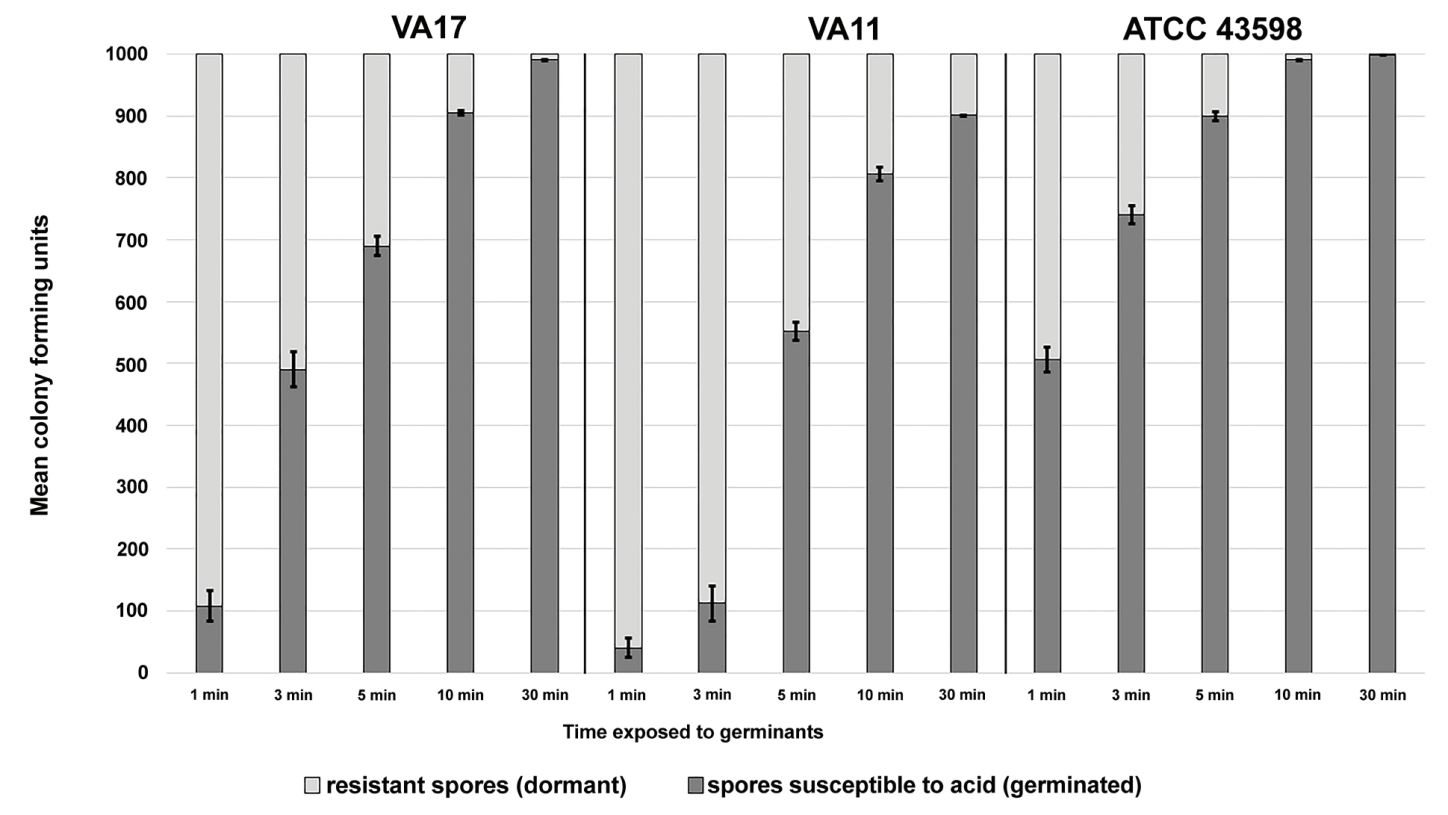
**Germinated spores are susceptible to killing by pH 1.5 hydrochloric acid.** One thousand colony forming units (CFU) of *C. difficile* VA17, VA11, and ATCC 43598 spores were exposed to 1 mL of germinants in a test tube for 1, 3, 5, 10, or 30 minutes under ambient conditions (22°C, aerobic). At the appropriate time point, spores were washed 3 times to remove germinants and then resuspended in 1 mL of pH 1.5 hydrochloric acid for 10 minutes. Spore suspensions were neutralized with phosphate-buffered saline and washed to remove hydrochloric acid. Contents were spread on a plate to enumerate the recovery of viable spores. The means of data from triplicate experiments are presented. Error bars indicate standard error.

In a real-world setting, the amount of time spores are exposed to germinants would be variable. Figure 6 shows that acid susceptibility is directly proportional to the amount of time the spores were exposed to germinants, and that the rates of germination were strain-specific. In our experience, approximately 3 log_10_CFU or less of *C. difficile* spores are commonly recovered from contaminated skin or environmental surfaces (author's unpublished data). For the 3 strains tested, more than 800 of 1000 spores were killed by acid after 10 minutes of exposure to germinants. After 30 minutes of exposure to germinants, acid killing was increased to 900 of 1000 spores for strain VA11, and more than 990 of 1000 spores for strains VA17 and ATCC 43598.

### Antibiotic-treated mice do not become colonized with C. difficile after exposure to germinated spores in their environment.

**Figure 7.**
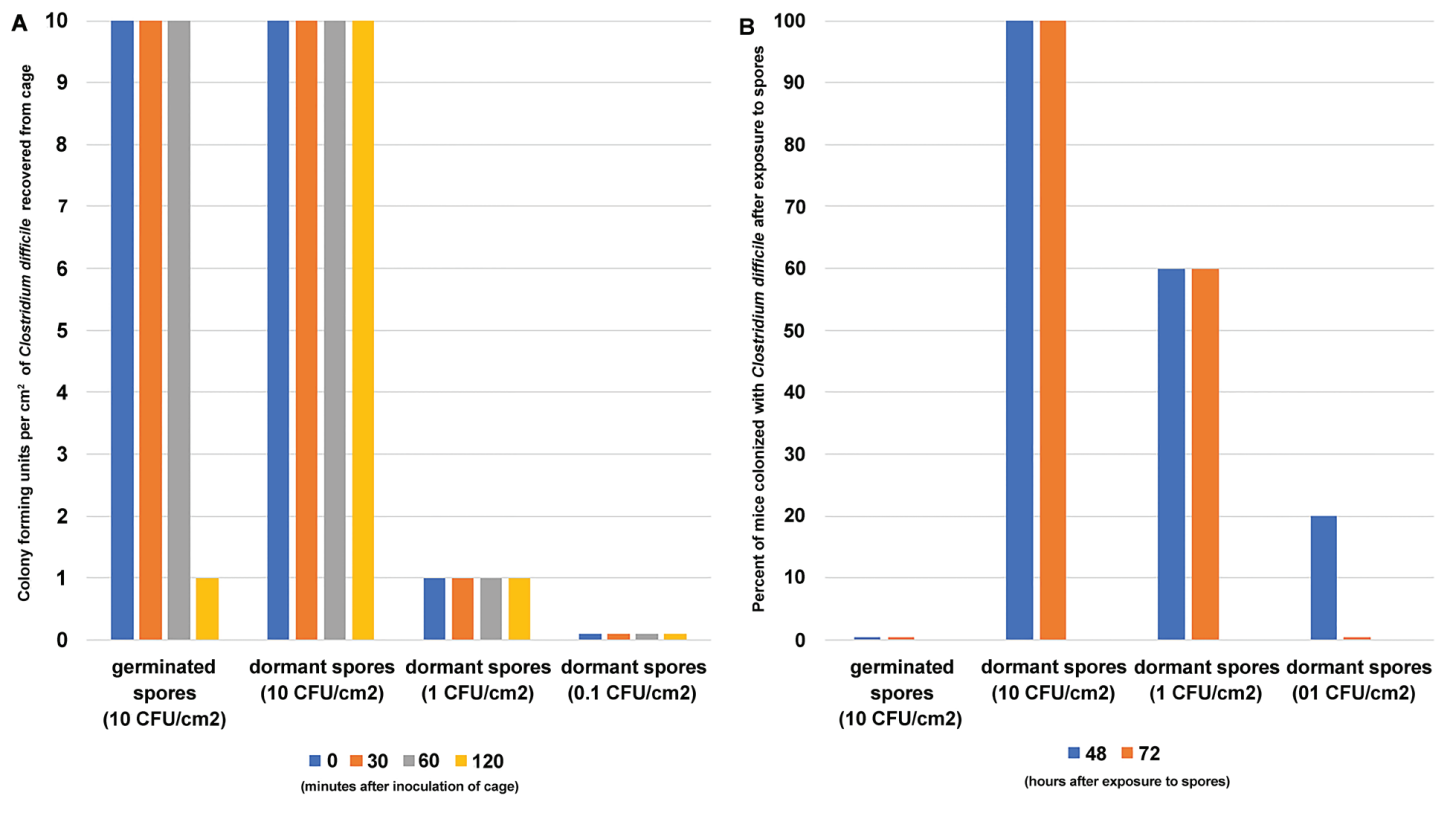
**Antibiotic-treated mice do not become colonized with *C. difficile* after exposure to germinated spores in their environment.** Cages were inoculated with either 10 CFU/cm^2^ germinated spores, 10 CFU/cm^2^ dormant spores, 1 CFU/cm^2^ dormant spores, or 0.1 CFU/cm^2^ dormant *C. difficile* spores. Antibiotic-treated mice were exposed to contaminated cages for 2 hours. Panel A shows the colony forming units of viable spores recovered from 1 cage at 0, 30, 60, and 120 minutes post inoculation. Panel B shows the percentage of mice that became colonized with *C. difficile* 48 and 72 hours after exposure to spores (N = 40 mice total, 20 mice 10 CFU/cm^2^ germinated spore group, 10 mice 10 CFU/cm^2^ dormant spore group, 5 mice 1 CFU/cm^2^ dormant spore group, and 5 mice 0.1 CFU/cm^2^ dormant spore group).

In a previously established murine model of *C. difficile* colonization, we demonstrated that mice treated with broad-spectrum antibiotics become susceptible to *C. difficile* colonization, whereas mice with intact microbiota do not [27, 28]. In this experiment, all the mice were treated with antibiotics to induce susceptibility to *C. difficile* colonization. Figure 7 shows that mice exposed to germinated spores in their environment (10 CFU/cm^2^) do not become colonized with *C. difficile*. However, 100% of mice exposed to the same inoculum of dormant spores (10 CFU/cm^2^) became colonized. When the environmental inoculum of dormant spores was reduced 10-fold (1 CFU/ cm^2^), 60% of the mice became colonized with *C. difficile*. Reducing the inoculum of dormant spores another 10-fold (0.1 CFU/cm^2^) decreased the percentage of mice colonized with *C. difficile* to 20%.

Germinated spores inoculated onto cage surfaces remained viable for 60 minutes, but after 120 minutes (full exposure time) the number of viable spores recovered from the cage dropped to 1 CFU/cm^2^. Dormant spores inoculated onto cage surfaces remained viable for the duration of the experiment.

## DISCUSSION

For the first time, we have demonstrated that *C. difficile* spores germinate on skin after a single application of cholic acid-class bile salts and co-germinants. There was strain-specific variability in the extent of germination, but for each strain tested the fraction of germinated spores correlated with a decrease in spore survival on skin. For solutions containing taurocholic acid, ethanol significantly enhanced reduction of viable spores on skin. On surfaces, exposure to the germinants in a high (70% v/v) or low (30% v/v) ethanol concentration matrix triggered germination within 10 minutes and reduced spore recovery. Germinated spores became susceptible to killing by pH 1.5 hydrochloric acid, suggesting that germinated spores that remain viable on skin and surfaces may be killed by acid on ingestion. Finally, antibiotic-treated mice did not become colonized after exposure to germinated spores, whereas 100% of mice became colonized after exposure to the same quantity of dormant spores.

Our findings have important practical applications. Induction of germination could provide a new and benign approach to reduce the burden of *C. difficile* spores on skin and in the environment. The fact that germinants were effective when combined with ethanol suggests that it may be feasible to modify existing hand sanitizers to develop alcohol-based sanitizers that reduce spores on the hands. According to the Material and Safety Data Sheets (MSDS) for l-glycine, calcium chloride, and bile salts, skin irritation may occur when exposed to the pure form of these reagents. However, further studies would have to be performed to test the effect of these reagents on skin at the low concentrations used for germinating spores. Germinants are stable at room temperature in solution and would be relatively inexpensive at the concentrations required. Similarly, alcohol-based surface disinfectants containing germinants could also provide a non-corrosive agent to reduce spores on surfaces. Finally, the fact that germinated spores did not establish colonization in mice suggests that use of exposure to germinants may decrease the likelihood of disease if spores are ingested.

Although the germination process has been well defined in *Bacillus* and other *Clostridia* spp., elucidation of *C. difficile* germination has only recently been initiated due to the lag in development of reliable and convenient molecular tools. Recent studies have revealed that *C. difficile* has a species-specific germination process that is triggered by bile salts and co-germinants [13]. While *Bacillus* and other *Clostridia* spp. employ inner membrane germinant receptors to sense small molecule nutrients that trigger dipicolonic acid (DPA) release and rehydration of the core, *C. difficile* uses a subtilisin-like serine protease as a germinant receptor to sense bile salts near the spore cortex [29]. As a result, degradation of the thick protective cortex layer precedes the release of DPA. These differences between *C. difficile* and other spore-forming bacteria have important implications for the use of germination as a means to enhance killing of *C. difficile* spores. Research is needed to identify and optimize chemical germinants that target *C. difficile's* unique mode of germination.

The microbiological basis for the increased susceptibility of germinated spores to a variety of stressors has not been clearly described. Our findings suggest that desiccation, and to a lesser extent the presence of oxygen, are the stressors primarily responsible for reductions of germinated spores on skin and surfaces. Additional studies are needed to identify other stressors that could enhance killing of germinated *C. difficile* spores in air at room temperature and that could explain their increased susceptibility to other stressors such as heat, UV light, and acid. De Sordi *et al* recently reported that triggering *C. difficile* spores to germinate with taurocholate enhanced the killing efficacy of photodynamic antimicrobial chemotherapy which uses light to treat infection of the colon [30]. In conjunction with our findings, such reports should stimulate research to identify other new methods to enhance the killing of germinated spores.

Our study has some limitations. The extent of germination varied in each of the 5 *C. difficile* strains tested. Thus, the effectiveness of this approach may vary for different strains. We tested the germinant solutions using an *ex vivo* porcine skin model; therefore, additional studies are needed in humans. The environmental surface testing was performed in a laboratory setting, and studies are needed in hospital rooms with natural *C. difficile* contamination because it is possible that factors such as organic material on surfaces could reduce the efficacy of the germinants. For the mouse colonization experiments, we isolated a fraction of spores that was > 99.99% germinated and demonstrated that these spores did not establish colonization. Our findings may over-estimate the ability of current germination approaches to reduce the ability of spores to colonize because a persistent “superdormant” fraction of spores remains unaltered by exposure to germinants. Ghosh and Setlow recently demonstrated that isolated superdormant spores of *Bacillus subtilis* and *Bacillus megaterium* require an increased signal for triggering spore germination compared to most spores in other populations [31]. Additional studies are needed to optimize germinants for *C. difficile* to further enhance killing by triggering germination in the superdormant fraction of spores. Finally, although our results suggested that germinated spores may be killed by stomach acid, further work is needed to determine the microbiological basis for the failure of germinated spores to establish colonization.
